# Perioperative period from a pediatric perspective: a mixed-methods study

**DOI:** 10.3389/fped.2025.1636208

**Published:** 2025-08-20

**Authors:** Selda Yüzer Alsaç, Gökçen Aydın Akbuğa

**Affiliations:** ^1^ Department of Pediatric Nursing, Faculty of Health Science, Yozgat Bozok University, Yozgat, Türkiye; ^2^ Department of Surgical Nursing, Faculty of Health Science, Yozgat Bozok University, Yozgat, Türkiye

**Keywords:** child, perioperative period, surgery experience, anxiety, mixed method

## Abstract

**Objective:**

To determine anxiety levels of children aged 8–12 years who were admitted to the pediatric clinic for surgery; to understand the anxiety and experiences in perioperative period, and to determine their physical and emotional needs in this process.

**Methods:**

The research was carried out in the surgical ward of a public hospital between October 2023 and February 2024. A mixed research method (quantitative and qualitative) was used. In the first phase, the State Anxiety Inventory for Children was administered to evaluate preoperative anxiety levels. In the second phase, in-depth interviews were conducted with children to explore their perceptions and experiences regarding the surgical process.

**Results:**

The mean age of the children was 9.72 ± 1.38 years, and 74.6% of the participants were boys (*N* = 126). The mean anxiety score of the children was 38.39 ± 10.56. It was detected that the mean anxiety score of younger children was higher (*p* < 0.000). The mean anxiety score of children who underwent genitourinary system surgery was found to be higher than other types of surgery (*p* < 0.035). It was also determined that the mean anxiety scores were lower among children who were informed before the operations decreased (*p* < 0.003). After in-depth interviews with children (*N* = 12), the themes of “The Effect of Hospital and Operating Room Environment”, “Social Support” and “Expectations from the Operating Room” were determined.

**Conclusion:**

The quantitative results of the study indicate that variables such as age, type of surgery, whether the child was informed about the surgery make a difference in children's anxiety levels. The qualitative results of the study revealed that children felt anxiety and fear about the hospital and surgery environment at every stage of the perioperative period.

## Introduction

Pediatric surgery, a branch of surgery, covers a wide age range of patients, from premature neonatals to adolescent children. Surgical interventions are a stressful and traumatic situation for children, especially before surgery, which can lead to high levels of anxiety (1). The hospital environment, staff, materials used, and procedures to be performed are a state of uncertainty for all groups of hospitalized children and may affect the child's adaptation to the hospital and treatment, communication with the healthcare professional, and future hospital experience. In addition to hospitalization, the fact that a surgical intervention will be performed causes more stress to be perceived by the child and causes the child to be affected more both psychologically and physiologically (2). Studies have reported that 50%–80% of children experience excessive anxiety before surgery (3–5). It has also been noted that most children with high levels of preoperative anxiety display behavioral changes in the postoperative period, recover more slowly, and experience moderate pain (1).

Children are particularly prone to preoperative anxiety due to their limited cognitive abilities and greater dependence on others (6). The separation of the child from home and family, strangers in the operating room and environment, the type of surgery and the waiting time increase the child's anxiety and worry (7). Adding the induction of anesthesia to these situations, behavioral problems such as depression, introversion, and increased dependency on the family may occur in the postoperative period and the need to take more analgesics (8). There are a number of factors that determine the child's reaction to this traumatic procedure and the unfamiliar hospital environment. These factors include the child's age, developmental level, personality structure, relationship with the family, previous experiences, economic and cultural status of the family, the attitude of the staff in the hospital, and the whole medical procedure (9). In pediatric surgery, preoperative preparations should be completed by the child's nurse, taking into account the child's age, developmental characteristics, and individual characteristics of the child and parents (10). Thus, the psychosocial effects of fear and anxiety that may be seen in children from the hospitalization process of the experience of having surgery and the physiological effects such as pain will be reduced (11). Several pieces of evidence indicate age and level of socioeconomic (9, 12), behavioral problems, previous surgery and hospitalizations (13, 14), level of parental education and maternal anxiety (15) as factors associated children with anxiety.

This study introduces an innovative approach to understanding the anxiety levels of children aged 8–12 years undergoing surgical procedures by focusing on their personal narratives and experiences before, during, and after surgery. By analyzing these descriptions, the research aims to uncover not only the psychological impact of hospitalization but also the specific physical and emotional needs of pediatric patients throughout the surgical process. This qualitative perspective allows for a more nuanced understanding of children's emotions and reactions, enabling healthcare providers to tailor interventions and support mechanisms that address their unique anxieties. Ultimately, the findings could lead to improved preoperative education, enhanced emotional support strategies, and a more child-centered approach to surgical care, fostering a more positive hospital experience for young patients. Additionally, literature reviews show a lack of studies in which both qualitative and quantitative research are conducted, showing how surgery preparation, the operating room, and postoperative social support affect children during the perioperative period, and reflecting factors related to preoperative child anxiety. This study aims to determine the anxiety level of children aged 8–12 years who are hospitalized for surgery based on their description of their experiences before, during or after surgery and to identify their physical and emotional needs throughout the process.

• What are the anxiety levels of children according to their descriptive characteristics before surgery?
• What are the physical, emotional and behavioral experiences of the children regarding the hospital environment and the operation?

## Methods

### Study design

**Figure 1:**
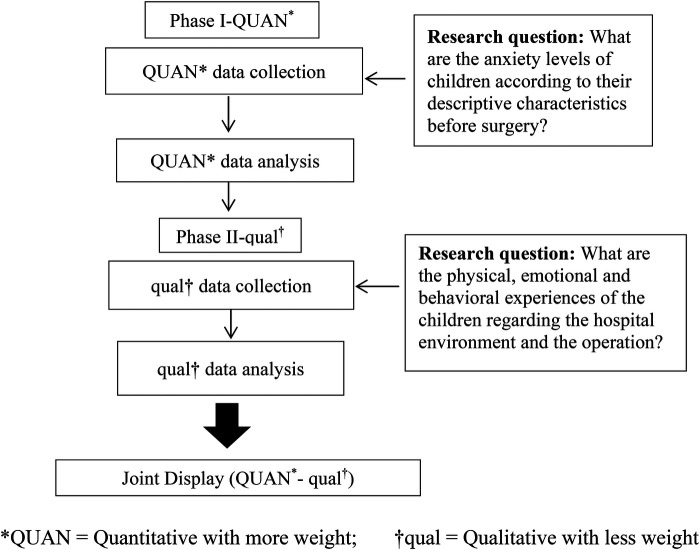
Representative diagram of the study design.

The research was carried out in the surgical ward of a public hospital between October 2023 and February 2024. This study adopted a mixed-methods approach and was conducted in two phases. The first phase focused on descriptive quantitative data aiming to determine the children's state anxiety levels just before the operation. Figure 1 shows the second phase focused on qualitative data obtained with a phenomenological approach to assess how children were affected by the hospital environment and the operation.

The inclusion criteria of the children in the study were determined as follows: The child must be in the age group of 8–12, hospitalization of the child for surgery, hospitalization of the child for more than one day and the child does not have a cognitive development problem.

### Phase one: descriptive quantitative data

Sample size calculations executed in G*Power 3.1 software, assuming a medium effect size of 0.3, a significance threshold of 5%, and a desired power of 95% (16). The first phase consisted of children aged 8–12 years who were hospitalized for surgery, met the inclusion criteria, and provided parental consent. “Demographic Information Form” and “State Anxiety Inventory for Children” were used for data collection. The demographic information form included questions compatible with the relevant literature (age, gender, diagnosis, reason for admission to the hospital, frequency of admission to the hospital, education level of the parents).

“State Anxiety Inventory for Children” (SAI) was developed by Spielberger to measure state and trait anxiety levels of children. In this study, a 20-item state anxiety scale was used. Its adaptation into Turkish, validity and reliability were conducted by Özusta (17). The state anxiety scale was created to determine how children feel at that moment. In this form, the options were scored as “not at all” (1), “a little” (2), and “a lot” (3). The highest score to be obtained from the State Anxiety Inventory is 60, and the lowest score is 20. An increase in the score indicates an increase in the level of anxiety. The Cronbach α value for the scale was found to be 0.82. In this study, cronbach *α* value was found to be 0.80. It took approximately 8–10 minutes to administer the scale. Information form data were collected at any time of the day the child was admitted to the clinic for surgery, and anxiety inventory data were collected at the entrance of the operating room just before the surgery.

### Phase two: phenomenological approach

The phenomenological method is a research approach to understand the essence of an event or experience. The main aim of this method is to discover the essence of individuals' thoughts, feelings, and perceptions (18). The COREQ (Consolidated Criteria for Reporting Qualitative Research) checklist was used in reporting the research (19). The phase 2 sample consisted of children who were willing to participate in the study and whose parents' consent was obtained. The volunteer sampling methfod was used for external validity. In the qualitative part of the research, data saturation was achieved and the sample size was deemed sufficient (20, 21). Data were collected using demographic information forms and semi-structured interview questions. Demographic Information Form consists of questions to collect information about the child. Semi-structured interview questions were developed after reviewing the relevant literature (22, 23). The following open-ended questions were asked to evaluate how children were affected by the operation.

*The semi-structured interview form questions:*

1. What does the hospital mean to you?
2. How did you feel when you were getting ready for the operation?
3. Who would you like to be with you when you were taken to the operation room?
4. How did you feel when you saw the objects in the operating room?
5. Was the operating room as you imagined it? How would you like it to be?
6. How did you feel after the surgery? Can you explain?

All interviews were conducted by the researcher (S.Y.A). Considering that children receive a sedative or analgesic medication that will affect their basic neurological status and emotional reactions, data from semi-structured questions were collected after the second or third day after surgery. The physiological status of the child was evaluated on the day of the interview.

Each interview lasted an average of 15–20 minutes using audio recording. After all the recordings were taken, the audio recordings were transcribed.

## Data analysis

### Analysis of the first phase

The SPSS 24.0 software was used to analyze the quantitative data in the first phase. Normality distributions were analyzed by Kolmogorov–Smirnov test and descriptive statistical analysis was performed by using number, percentage, mean, and standard deviation. Parametric test, one-way analysis of variance (ANOVA) and independent groups *t*-test (student *t*-test) were applied.

### Analysis of the second phase

In the second phase, Colaizzi's seven-step data analysis method was used to analyze the qualitative data (24). These steps are given below respectively:

1. Reading all participants' responses and determining the general meaning,
2. Extracting key statements from the answers that address the research,
3. Determining the meaning for key expressions,
4. Grouping the meanings of key expressions into theme clusters,
5. Combining the themes in a description of the researched topic,
6. Formulating the explanations as a precise statement that defines all their meanings; and
7. Validation with participants.

In the analysis of the second phase, one researcher (G.A.A.) read the transcripts several times. Then, all authors identified important statements about the children's experiences in the transcripts. The researcher formulated the key phrases that emerged from these important statements and coded a total of 100 formulated meanings. The meanings were categorized into three themes and six sub-themes. Then, the statements about children's operating room experiences were synthesized and explained. Finally, as the mothers were with the children during the interviews, the mothers of the children were contacted to confirm the accuracy and meaning of the statements.

The number of participants contributing to each theme/subtheme is provided below:

Theme 1: Six children contributed to the theme “The Effect of Hospital and Operating Room Environment”. (Fear and Anxiety sub-theme: 3 children, Social Isolation sub-theme: 3 children).
Theme 2: Seven children contributed to the theme “Social Support.” (Support of Family and Medical Staff sub-theme: 4 children, Routine and Familiarity sub-theme: 3 children).
Theme 3: Six children contributed to the theme “Expectations from the Operating Room.” (Supportive Environment sub-theme: 3 children, Physical and Emotional Comfort sub-theme: 3 children).

### Reliability

The reliability of the data was verified based on Colourafi and Evans' strategies such as objectivity, credibility, reliability, transferability, and applicability (25). At the beginning of the interview, a researcher (S.Y.A) encouraged the child's experiences to be expressed freely. All of the children's expressions were recorded with a voice recorder to increase the internal reliability (consistency) of the research and given directly in the findings section.

• The external reliability of the research was confirmed by submitting all data collection tools, raw data, the coding made during the analysis phase, and the perceptions, notes, and inferences that form the basis of the report to the examination of a faculty member from the Department of Sociology.
• Content analysis was conducted to increase the internal validity (credibility) of the research. The opinion of an expert academic not involved in the study and having experience in qualitative studies was consulted. The integrity was provided by checking the relationship between the three conceptual themes and two sub-themes and the relationship of each theme with the others.
• The research process was explained in detail to ensure the external validity (transferability) of the research. Detailed information about the characteristics of the participants, the research model, the purpose of the research, the study group, the data collection tool, the inclusion criteria, and the method was given. Data analysis and interpretation were described in detail. Before the interview, the researcher introduced himself, gave information about the purpose of the research, and tried to gain the trust of the participant so that the data collected during the interview process reflected the real situation.

### Ethical considerations

Ethical approval was obtained from Yozgat Bozok University Ethics Committee (40/04:2022) along with institutional permission to conduct the study. Parents and children were informed about the purpose and content of the study, and written and verbal consent was obtained from the parents in case of participation. Children's names and data were kept confidential (audio recordings and transcriptions were kept in a safe place, children's names were removed from all documents, and codes were assigned. For example: C1, C2, C3).

## Results

### Phase 1 results

Table 1 reflects the first phase of the study. The mean age of the children was 9.72 ± 1.38 years, and 74.6% were boy. It was found that 37.3% of the children had ENT (Ear, Nose and Throat) surgery, and 47.6% were informed before surgery.

**Table 1:** Descriptive characteristics of children (*n* = 126).

Characteristics	*N*	%
Gender		
Girls	32	25.4
Boys	94	74.6
Type of surgery		
ENT (ear, nose and throat)	47	37.3
Orthopedics	41	32.5
Genito-urinary	25	19.8
GIS (gastro-intestinal system)	13	10.3
Preoperative information status		
Yes	60	47.6
No	66	52.4
Knowing that the operation will be performed		
Yes	98	77.8
No	28	22.2
Mother's educational status		
Elementary Education	61	48.4
High school	50	39.7
University and above	15	11.9
Father's educational status		
Elementary Education	24	19.0
High school	73	57.9
University and above	29	23.1
M^a^ ± SD^b^		
The average age of the child	9.72 ± 1.38	
Average age of mother	35.17 ± 3.94	
Average age of father	36.85 ± 3.43	

^a^ M, mean.

^b^ SD, standard deviation.

Table 2 illustrates the comparison between the characteristics of the children, and their mean state anxiety scores. The mean anxiety score of the children was 38.39 ± 10.56. It was detected that the mean anxiety score of younger children (age 8) was higher than that of older children and the difference was statistically significant (*p* < 0.000). The mean anxiety score of children who underwent genitourinary system surgery was found to be higher than other types of surgery, and the difference was statistically significant (*p* < 0.035). It was also determined that the mean anxiety scores of children who were informed before the operation decreased, and the difference was statistically significant (*p* < 0.003). The mean anxiety scores of the children whose fathers were primary school graduates were higher than the other children, and the difference was statistically significant (*p* < 0.001). In the study, no significant difference was found between the mean anxiety score and gender of the child, knowledge that the surgery would be performed, and the educational status of the mother and the scale.

**Table 2:** Comparison of children's descriptive characteristics and mean scores of “state anxiety inventory for children” (*n* = 126).

Features		*n*	SAI^c^	
			M^a^ ± SD^b^	Test/p
Mean anxiety score			38.39 ± 10.56	
Age	8 years old	33	42.48 ± 11.61	*F*: 6.027
	9 years old	26	42.26 ± 09.03	***p*: 0.000^d^**
	10 years old	27	38.25 ± 10.19	*η*²: 0,166
	11 years old	23	31.82 ± 06.69	
	12 years old	17	33.64 ± 09.96	
Gender	Girls	32	37.31 ± 10.83	*t*: −0.660
	Boys	94	38.76 ± 10.50	*p*: 0.512^e^
Type of surgery	Genito-urinary	25	41.24 ± 11.55	
	ENT (ear, nose and throat)	47	38.65 ± 09.54	*F*: 2.673
	Orthopedics	41	38.60 ± 10.29	***p*: 0.035** ^d^
	GIS (gastro-intestinal system)	13	31.30 ± 11.02	*η*²: 0,062
Preoperative information status	Yes	60	35.46 ± 12.01	*t*: −3.066
	No	66	41.06 ± 08.28	***p*: 0.003** ^e^
				Cohen's *d*: 0.54
Knowing that the operation will be performed	Yes	98	39.07 ± 10.93	*t*: 1.506
	No	28	36.03 ± 08.92	*p*: 0.138^e^
Mother's educational status	Elementary Education	61	40.36 ± 10.22	*F*: 2.715
	High school	50	37.34 ± 10.96	*p*: 0.070^d^
	Above University	15	33.93 ± 09.26	
Father's educational status	Elementary Education	24	44.08 ± 09.65	*F*: 7.862
	High school	73	38.61 ± 10.29	***p*: 0.001** ^d^
	Above University	29	33.13 ± 09.63	*η*²: 0,123

Bold values indicate significant *p* < 0.05.

^a^ M, mean.

^b^ SD, standard deviation.

^c^ SAI, state anxiety inventory.

^d^ One-way analysis of variance (ANOVA).

^e^ Independent groups *t*-test (student t-test) η², cohen's *d* effect size

### Phase 2 results

Table 3 shows that eight of the children (%66.7) were male, and one-third of the children were operated on because of an arm fracture (33.3%). Table 4 presents, as a result of the analysis of the statements of the children obtained with the semi-structured interview form, three main themes and two sub-themes related to each theme were determined.

**Table 3:** Descriptive characteristics of children (*n* = 12).

Child	Age	Gender	Medical Diagnosis
C 1	8	Boy	Phimosis
C 2	9	Boy	Testicular surgery
C 3	10	Boy	Inguinal hernia
C 4	8	Girl	Arm fracture
C 5	10	Girl	Ganglion Cyst
C 6	8	Boy	Phimosis
C 7	12	Boy	Ankle Fracture
C 8	10	Boy	Adenoidectomy
C 9	11	Girl	Arm fracture
C 10	12	Boy	Arm fracture
C 11	11	Girl	Arm fracture
C 12	8	Boy	Adenoidectomy

**Table 4:** Themes and sub-themes of the research.

Theme	Sub-Theme
The Effect of Hospital and Operating Room Environment	• Fear and anxiety • Social Isolation
Social Support	• Support of Family and Medical Staff • Routine and Familiarity
Expectations from the Operating Room	• Supportive Environment • Physical and Emotional Comfort

**Theme 1. The Effect of Hospital and Operating Room Environment:** In this theme, children expressed their fears and anxieties about the surgery and the postoperative period, and their experiences of social isolation.

**Sub-theme 1.1. Fear and Anxiety:** Most of the children expressed fear of being separated from their parents, feeling pain, and being afraid of medical devices and instruments. Children were also anxious because they did not fully understand what the surgery was and why it would be performed and because they were in a foreign environment.

*C1: The hospital is very scary; I am afraid of the needle, and I am also afraid of the nurse and the doctor. In case they drill me…*

*C5: When I was getting ready for the surgery, I was scared to wear a gown. My heart pounded a little bit, so I closed my eyes. I thought a little bit about how they would do it because it was the first time I was going to have surgery..I thought about how long I would be separated from my brother…*

*C8: I was very scared and panicked when they put me to bed. My heart pounded a little bit, so I closed my eyes, and they took the adenoids, and then I opened my eyes.*

**Sub-theme 1.2. Social Isolation:** Children stated that they felt lonely because they were separated from school, friends, and family members.

*C3: I want to go home; I am very bored here; I miss my brother; he and I are alone; either my father is with me or my mother. It's better to be together…*

*C7: I am happy that the pain in my wrists is gone, but I can't stand up. I can't go to school, I can't play soccer; I am all alone.*

*C8: The hospital is not a place for children; it is too boring, I cannot play with anyone; everyone is an adult; my mother does not allow me to take care of other children; I sleep alone.*

**Theme 2. Social Support:** In this theme, children revealed the meaning of emotional support from family and healthcare staff, routines established in the hospital, and the presence of familiar people and objects in the environment during the surgery process.

**Sub-theme 2.1. Support of Family and Medical Staff:** Children expressed that they felt safe having a family member with them on the way to the surgery and throughout the treatment process, as well as having healthcare staff with them, and that their anxiety decreased when communicating with them. Moreover, most of the children were remarkably focused on the presence of the nurse and her explanations during the surgical transfer and expressed their feelings at the moment of transfer more clearly during the interview.

*C3: Doctors and nurses prepared me for the surgery. At first I was very scared, but someone with a hat and a mask on her head, I think it was a female nurse, told me not to be afraid. She told me that the surgery would be over in a short time and I would return home in good health. So my fear decreased a little bit.*

*C6: My mother and the female nurse took me to the surgery. My mom held my hand, and my fear lessened. The female nurse joked with me because I was going to be circumcised; it was funny. I wish they were inside..I wish my father was there.*

*C9: My mother goes everywhere with me and protects me from strangers. My brother also had surgery and he told me not to be afraid, everything will be fine, and you will recover and come back. I just got through it…*

*C10: On the way to the operation, the nurse asked me if I was scared. As if she wouldn't be scared if it was her own child? We chatted for a while as her child was also undergoing surgery. The next thing I knew, I walked in, and there was a huge light. I didn't understand at all.*

**Sub-theme 2.2. Routine and Familiarity:** Children stated that their families and healthcare staff explaining certain routines and following these routines and having people they know or items they brought from home, such as toys and books, in the environment made them feel safe and good.

*C9: The female nurse came in the morning; she always takes care of me; she brought me a gown; she made me wear it and then she told me, “I will take you to the surgery, and I will pick you up after you get out, and we will meet again”. I was relieved to see her; I think she was waiting for me at the door…*

*C10: I wish Mario Gómez (a German soccer player) was in my surgery. I always dream of meeting him. Seeing him makes me very happy. It makes me very happy to see him.*

*C11: After the operation, I wanted to come to my room immediately. I brought this toy that I love very much (showing it…), I would be sad if it stayed here. When I hug it, it understands me.*

**Theme 3. Expectations from the Operating Room:** In this theme, children discussed their expectations by expressing the operating room environment they imagined and the negative situations they experienced in the operating room.

**Sub-Theme 3.1. Supportive Environment:** Most of the children felt that the operating room was not a place for children. They stated that the operating room should be painted with colorful paints, cartoon characters could be on the walls, and they should be able to watch cartoons or play games in the operating room.

*C2: Everyone gets scared when they see the operating room; I think it is very scary. If all the objects were colorful, it (the operating room) could look beautiful like a rainbow. Also, if there were heroes I liked, for example, Spiderman, Batman, PJ Masks. I would look at them and not be afraid.*

*C5: If doctors and nurses were cartoon heroes, no child would be scared, and it would be fun. They can wear colorful masks. King Şakir and Necati (cartoon heroes) should also have pictures.*

*C6: If I had a tablet or played games on my phone during the circumcision, I would never think about it…*

**Sub-Theme 3.2. Physical and Emotional Comfort:** Some of the children expressed that they felt very cold in the operating room; there were exaggerated lights; they heard strange noises; the instruments were strange; and they felt uncomfortable.

*C1: There was a hall like this (describing the operating room); it was very big; there was a lot of equipment around; it was very complicated; the thing I was lying on was very cold; I felt cold; there were huge lights; my eyes felt strange when I entered.*

*C3: I was scared when I entered; there were voices, but a brother spoke to me. He was masked. He showed me some tools and said, “Look, it looks like a toy, doesn’t it?” We chatted, I counted…Then I felt like I woke up. I wonder where that brother is? I would like to thank him…*

*C4: Have you ever seen it? (Turning to the researcher and asking about the operating room). It is very cold, there are a lot of people you don't know, there are instruments, but everything is clean; everything is the same color. I don't want to go there again; I got bored there…*

## Discussion

Preoperative anxiety in children is strongly linked to postoperative pain, waking delirium, and behavioral changes (such as general anxiety, loss of appetite, sleep disturbances, incontinence, and sudden outbursts of anger) (26). Therefore, it is vital that anxiety is managed effectively. Possible causes of preoperative anxiety include child-related factors such as the child's age, health status, type of operation, separation from parents, unfamiliar surroundings, noise from the surgical team, fear of the unknown, and lack of support (27, 28).

The preoperative anxiety level of the children who participated in our study was 38.39 ± 10.56. In our qualitative findings, most of the children stated that they were afraid of separation from their parents, feeling pain, medical devices, and instruments. Furthermore, the children were anxious because they could not fully comprehend what the surgery meant and why it needed to be performed, and because they were in a foreign environment, separated from school, friends, and family members.

It was argued that the age of the child was an important factor among the factors causing anxiety. The age of a child was a crucial factor in understanding and addressing anxiety because it influenced their cognitive development, emotional regulation, and social interactions. For this reason, younger children might have lacked the developmental tools to articulate their fears or understand the sources of their anxiety, often expressing distress through behavior rather than words (27). In our study, younger children (age 8) had higher levels of anxiety. Likewise, in a study examining the postoperative psychosocial symptoms of children aged 6–12 years, it was determined that the anxiety scores of younger children (age 6 and 7) were higher than those of children in other age groups (29). Moura et al. (2016), found that the anxiety level of children in an age group of 5–6 years was higher than that in an age group of 7–12 years (9).

Moreover, the preoperative anxiety level of children undergoing genito-urinary system surgery was found to be higher than that of children undergoing ENT, orthopedics, and GIS surgery in our study. An analysis of the findings revealed that the younger age group consisted of most of the children (64.0%) who underwent genito-urinary tract surgery. This could have been explained by the fact that children in the younger age group generally do not have fully developed abstract thinking skills, had difficulty understanding complex processes such as surgery, felt more insecure in unfamiliar environments, had inadequate anxiety management skills, and tended to be dependent on their parents (6).

In our study, it was determined that children who were not informed before surgery had high anxiety scores. Moreover, some of the children stated that their fears decreased by focusing on the explanations made by the nurse during the surgical transfer and in the operating room. In parallel with the results of our study, Shaheen et al. reported that age-appropriate information about anesthesia and the surgical process reduced preoperative anxiety and increased cooperation (30). In another study, it was argued that audiovisual training on preanesthetic information in children was an effective approach to reduce preoperative anxiety (28).

Studies revealed that preoperative information and education were important in reducing children's anxiety. It was thought that all children, regardless of age and developmental level, needed physical, emotional, and cognitive preparation for surgery in the preoperative period. In the perioperative period, the period when children experienced the most intense fear and anxiety was the moment of hospitalization, the night before the day of surgery, the moment of transfer, the time when premedication drugs were administered, and the moment of anesthesia induction (31). In the studies, some non-pharmacologic methods related to these processes were recommended to reduce preoperative anxiety. These methods included parental presence, distraction techniques, entertaining transportation systems, preoperative information programs, hypnosis, and music therapy (32, 33). In the qualitative results of our study, the children reported that they felt confident that they were accompanied by a family member as well as healthcare personnel on the way to the surgery and throughout the treatment process, and that their anxiety decreased while communicating with them. Furthermore, the children stated that the fact that their families and healthcare personnel explained certain routines and that these routines were followed, and that the presence of people they knew or items they brought from home, such as toys and books, made them feel safe and good. Children's statements were in line with the literature.

In our study, children expressed their expectations about the operating room and the process in a striking way. Most of the children felt that the operating room was not a place for children. They stated that the operating room should have been painted with colorful paint, cartoon characters could have been on the walls, and they should have been able to watch cartoons or play games in the operating room. An examination of the literature (34–37) showed that practices parallel to children's expectations were successful in managing anxiety and decreased postoperative negative behavioral changes. In their study, Vagnoli et al. stated that clowns who interacted with children before entering the operating room and stayed with children with their parents during the anesthesia induction procedure reduced children's anxiety levels (38). Another study determined that music therapy and kaleidoscope distraction methods yielded positive results to reduce pain intensity, fear, and anxiety in the postoperative period in circumcised children (33). In a study conducted in children aged 3–7 years, play dough was found to reduce preoperative anxiety (39). With the dissemination of these practices, it could be thought that children's expectations regarding the process would be met and anxiety would be reduced**.**

In our study, some of the children stated that they felt very cold in the operating room; there were exaggerated lights; they heard strange sounds; the instruments were strange; and they felt uncomfortable. One of the children expressed the situation as follows: “…*It was very big; there was a lot of equipment around; it was very complicated; the thing I was lying on was very cold; I felt cold; there were huge lights; my eyes felt strange when I entered”.* Another child stated that a familiar object emotionally comforted her, saying “*I wanted to come to my room immediately after the surgery. I brought this toy that I love very much (showing it…) I would be sad if it stayed here”. She stated that a familiar object emotionally comforted her as “she understood me when I hugged her”*. The physical and emotional comfort theme of the study was related to these statements.

In a study in which interventions to provide physical and psychospiritual comfort were implemented in parallel with the children's statements, it was found that the anxiety and fear levels of children in the surgical process who received comfort-oriented nursing care based on comfort theory were lower than standard care. In the study, it was suggested that comfort-oriented care would potentially contribute to improving health outcomes and ensuring the sustainability of care quality and care (40). It is thought that comfort-oriented care in the perioperative process will be effective in providing physical and emotional comfort to children and reducing anxiety levels.

## Limitations and strengths

This study included the experiences of children about the surgery they underwent. The limitations of the study were that children were not as competent as adults in verbalizing their experiences about the surgery, the answers given to the questions asked were scattered, the subject was interrupted, and most of the children participating in the study were male.

The strength of our study lies in evaluating childdren' perspectives themselves on the experience of surgery environment. In addition, it is the combination of both qualitative and quantitative methods in a study involving children.

## Conclusions and recommendations

The quantitative results of the study indicate that variables such as age, type of surgery, whether the child was informed about the surgery, and parental education level make a difference in children's anxiety levels. It was determined that the mean anxiety scores of younger children were higher, the anxiety levels of children who underwent genitourinary surgery increased, informing children about the surgery before surgery decreased their anxiety scores, and low parental education level increased the mean anxiety score of the child. The qualitative results of the study revealed that children felt anxiety and fear about the hospital and surgery environment at every stage of the perioperative period and showed physiological and psychosocial reactions. Children expressed that cartoon characters should be used in all areas of the operating room, the operating area and its surroundings should be colorful, the health personnel should have clothes that are remarkable, colorful, and consisting of cartoon characters, and one of the family members should be with the child.

The perception of anxiety in the pediatric group also depends on the developmental stage and cognitive potential of the child, and different reactions may be observed among those who face the same stressor. Therefore, education and information should be provided, taking into account the age and developmental capacity of each child. In addition to this,

• children should be provided with a quiet and comfortable environment, the use of special guidance methods that will include family members,
• the caregiver should be fixed and informed consent should be given in a language that the child understands, the child's
• accompanied by a fun game for transfer, and if possible, one of
• the parents should accompany the child during the intraoperative period,
• introducing the mayo table and operating room in the intraoperative period,
• coloring the operating rooms where pediatric surgery is performed,
• making the uniforms of the health personnel from cartoon characters,
• and ensuring that the child meets with family members (brother, sister, sibling, father) during the process can significantly reduce children's perioperative period anxiety and thus increase their adaptation to the hospital and the disease.

## Data Availability

The original contributions presented in the study are included in the article/Supplementary Material, further inquiries can be directed to the corresponding author.
